# A sequence-based hybrid predictor for identifying conformationally ambivalent regions in proteins

**DOI:** 10.1186/1471-2164-10-S3-S22

**Published:** 2009-12-03

**Authors:** Yu-Cheng Liu, Meng-Han Yang, Win-Li Lin, Chien-Kang Huang, Yen-Jen Oyang

**Affiliations:** 1 Institute of Biomedical Engineering, National Taiwan University, Taipei, Taiwan, Republic of China; 2 Department of Computer Science and Information Engineering, National Taiwan University, Taipei, Taiwan, Republic of China; 3 Graduate Institutes of Biomedical Electronics and Bioinformatics, National Taiwan University, Taipei, Taiwan, Republic of China; 4 Department of Engineering Science and Ocean Engineering, National Taiwan University, Taipei 106, Taiwan, Republic of China; 5 Center for Systems Biology and Bioinformatics, National Taiwan University, Taipei, Taiwan, Republic of China

## Abstract

**Background:**

Proteins are dynamic macromolecules which may undergo conformational transitions upon changes in environment. As it has been observed in laboratories that protein flexibility is correlated to essential biological functions, scientists have been designing various types of predictors for identifying structurally flexible regions in proteins. In this respect, there are two major categories of predictors. One category of predictors attempts to identify conformationally flexible regions through analysis of protein tertiary structures. Another category of predictors works completely based on analysis of the polypeptide sequences. As the availability of protein tertiary structures is generally limited, the design of predictors that work completely based on sequence information is crucial for advances of molecular biology research.

**Results:**

In this article, we propose a novel approach to design a sequence-based predictor for identifying conformationally ambivalent regions in proteins. The novelty in the design stems from incorporating two classifiers based on two distinctive supervised learning algorithms that provide complementary prediction powers. Experimental results show that the overall performance delivered by the hybrid predictor proposed in this article is superior to the performance delivered by the existing predictors. Furthermore, the case study presented in this article demonstrates that the proposed hybrid predictor is capable of providing the biologists with valuable clues about the functional sites in a protein chain. The proposed hybrid predictor provides the users with two optional modes, namely, the *high-sensitivity *mode and the *high-specificity *mode. The experimental results with an independent testing data set show that the proposed hybrid predictor is capable of delivering sensitivity of 0.710 and specificity of 0.608 under the *high-sensitivity *mode, while delivering sensitivity of 0.451 and specificity of 0.787 under the *high-specificity *mode.

**Conclusion:**

Though experimental results show that the hybrid approach designed to exploit the complementary prediction powers of distinctive supervised learning algorithms works more effectively than conventional approaches, there exists a large room for further improvement with respect to the achieved performance. In this respect, it is of interest to investigate the effects of exploiting additional physiochemical properties that are related to conformational ambivalence. Furthermore, it is of interest to investigate the effects of incorporating lately-developed machine learning approaches, e.g. the random forest design and the multi-stage design. As conformational transition plays a key role in carrying out several essential types of biological functions, the design of more advanced predictors for identifying conformationally ambivalent regions in proteins deserves our continuous attention.

## Background

Proteins are dynamic macromolecules which may undergo conformational transitions upon changes in environment, such as pH, temperature, or upon interactions with other macromolecules [1]. It has been observed in laboratories that conformational transition plays a key role in carrying out several essential types of biological functions, including enzyme catalysis, macromolecule recognition, binding, and signal transduction [2]. For instance, the GTPase HRas protein, whose gene serves as an oncogene of the bladder cancer, shows different conformations in the Switch II region when this protein switches between the RAS-GTP state and the RAS-GDP state [3-6]. Another example is the U1 snRNP A from Homo sapiens. The conformation of one portion of the RNA binding region switches from a helix in the unbound state to a loop in the bound state respectively [7,8]. Conformational switches sometimes even cause diseases. For instance, the prion protein (PrP) causes the mad cow disease when a specific secondary structure element changes from a helix to a *β*-sheet [9].

As conformational flexibility is related to protein functions and interactions, scientists have been designing various types of predictors for identifying conformationally flexible regions in proteins [10-12]. In this respect, there are two major categories of predictors. The problem that was firstly addressed by Young et al. [12] concerns identifying polypeptide segments that may fold to form different secondary structure elements in different environments based on sequence analysis. Another major category of the predictors attempts to identify conformationally flexible regions through analysis of protein tertiary structures [2]. As the availability of protein tertiary structures is generally limited, the design of predictors that work completely based on sequence information is crucial for advances of molecular biology research.

In this article, we will propose a novel approach to design a sequence-based predictor for identifying conformationally ambivalent regions in proteins. The novelty in the design stems from incorporating two classifiers based on two distinctive supervised learning algorithms that provide complementary prediction powers. These two machine learning algorithms are the relaxed variable kernel density estimation (RVKDE) algorithm [13,14] and the QUICKRBF algorithm [15] that we have recently proposed. With these two classifiers, the proposed hybrid predictor can operate under either the *high-sensitivity *mode or the *high-specificity *mode, depending on the user' s application. Experimental results show that the overall performance delivered by the proposed hybrid predictor is superior to the performance delivered by the existing predictors. Furthermore, the case study presented in this article demonstrates that the proposed hybrid predictor is capable of providing the biologists with valuable clues about the functional sites in a protein chain.

## Results

### Overview of the proposed hybrid predictor

**Figure 1:**
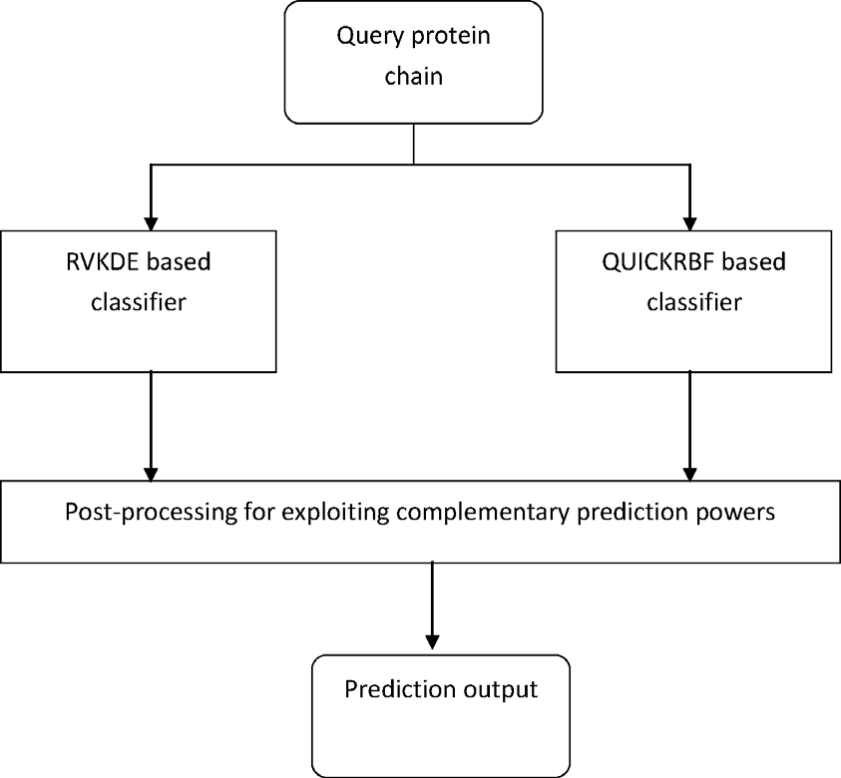
**The overall structure of the proposed hybrid predictor**.

Fig. 1 presents an overview of the structure of the proposed hybrid predictor. The hybrid predictor consists of two classifiers that have been constructed based on two distinctive supervised learning algorithms. As mentioned earlier, the motivation to incorporate two distinctive classifiers was to exploit the complementary prediction powers of alternative supervised learning algorithms. During our study, we observed that for the application addressed in this article the QUICKRBF based classifier consistently delivered sensitivity around 0.7 and specificity around 0.6 regardless of how the user-controlled parameter was set. Aiming to achieve higher specificity, we therefore investigated the possibility of incorporating two distinctive classifiers with complementary prediction powers. Our study ended up with the design shown in Fig. 1. When the hybrid predictor operates under the *high-sensitivity *mode, only the QUICKRBF based classifier is enabled. On the other hand, when the hybrid predictor operates under the *high-specificity *mode, both classifiers are enabled and their outputs are merged to achieve higher specificity. With respect to merging the outputs of two classifiers, the following mechanism has been employed. In case the RVKDE based classifier predicts a residue to be conformationally ambivalent but the QUICKRBF based classifier makes an opposite prediction, then the hybrid predictor will check the predictions made by the QUICKRBF based classifier for the four adjacent residues. If three out of the four adjacent residues are marked as conformationally ambivalent, then the concerned residue will be marked as conformationally ambivalent as well. Otherwise, it will be marked as conformationally rigid. Similarly, in case the QUICKRBF based classifier predicts a residue to be conformationally ambivalent but the RVKDE based classifier makes an opposite prediction, then the hybrid predictor will check the predictions made by the RVKDE based classifier for the four adjacent residues. If three out of the four adjacent residues are marked as conformationally ambivalent, then the concerned residue will be marked as conformationally ambivalent as well. Otherwise, it will be marked as conformationally rigid.

The basis of the mechanism described above for merging the outputs of the QUICKRBF based classifier and the RVKDE based classifier is to adopt a more cautious stand in predicting a residue to be conformationally ambivalent. During our study, we tried several alternative mechanisms and decided to employ the one describe above due to its effects observed in the cross validation procedure. The detailed design of QUICKRBF based classifier and the RVKDE based classifier as well as the cross validation procedure employed to set the parameters of the classifiers will be elaborated in the section entitled " Methods".

### Generation of the training data set

Both the learning processes of the RVKDE based classifier and the QUICKRBF based classifier in Fig. 1 have been carried out with the training data set generated by the following procedure.

**Figure 2:**
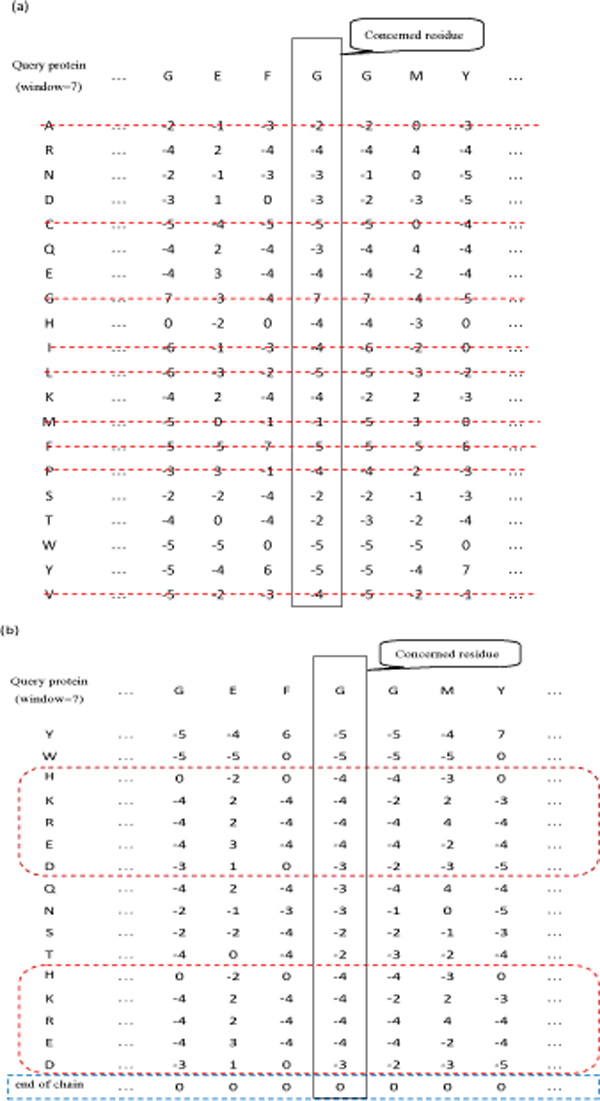
**An illustration of the process employed to generate the feature vector of the residue of concern**. (a) The feature vectors are derived from PSSM with window size set to 7. Rows corresponding to residue types that are neither charged nor polar are deleted. (b) Rows corresponding to residue types with charge are duplicated and one additional row is included to indicate whether the residue is at one end of the protein chain or not.

(1) All the protein chains in the PDB [16] (released on 01-April-2008) that have the same entry name and primary accession number in SwissProt (release 55.1 of 18-March-2008) are grouped. In the end, there are a total of 11084 groups of protein chains.

(2) The BLAST package [17] is invoked to check the redundancy among the groups of protein chains. It is guaranteed that no two protein chains in different groups have a sequence identity higher than 25%. In the end, 3496 groups of protein chains remain.

(3) For each of the 3496 groups of protein chains, the CLUSTALW package [18] is invoked to carry out multiple-sequence alignment on the protein chains in the group and the DSSP package [19] is invoked to label each residue in the protein chains with one of the following 3 types of secondary structure: helix, sheet, and coil. Then, one protein chain is randomly selected from each group as the representative. In this respect, we further checked the sequence identity between the 3496 representatives and the collection of 170 testing protein chains described in the next subsection. We removed 92 representatives due to there existing a homologous testing protein chain with a BLAST-computed sequence identity higher than 20%. Finally, each residue in the remaining 3404 representative protein chains was examined to determine whether the residue and all the residues in other protein chains that are aligned with the residue have been labelled with the same type of secondary structure. A conformationally ambivalent region is defined to be a segment of 3 or more consecutive residues within which each residue and the aligned residues have discrepant types of secondary structures.

(4) The training data set is generated by associating each residue in the 3404 representative protein chains with a feature vector. The feature vectors are derived from the position specific scoring matrices (PSSM) computed by the PSI-BLAST package [20] with window size set to 7. As illustrated in Fig. 2(a), those rows in the PSSM that correspond to residue types that are neither charged nor polar are deleted. Furthermore, as illustrated in Fig. 2(b), those rows corresponding to residue types with charge are duplicated to increase their influence. Then, a new row is added to record whether the corresponding position is at one end of the protein chain. Finally, the feature vector is generated by concatenating all the rows in the matrix and the values in the feature vectors are scaled to range from 0 to 1 by applying the standard logistic function:

One may wonder why we discarded those rows in the PSSM that correspond to residue types that are neither charged nor polar. The reason was that we conducted an analysis on the propensity of residue types in conformationally ambivalent regions and found that the propensity of hydrophobic residues is essentially uniform in conformationally ambivalent regions and in rigid regions. On the other hand, the conformationally ambivalent regions contain significantly higher percentage of charged and/or polar residues than rigid regions. Therefore, we duplicated those rows in the PSSM that correspond to residues with charge.

### Generation of the independent testing data set

The experiments reported in this article have been conducted with an independent testing data set derived from the collection of protein conformational ambivalence regions created by Boden et. al. http://pprowler.itee.uq.edu.au/sspred/[10]. According to Boden' s description, this collection of 170 protein chains was extracted from MolMovDB http://www.molmovdb.org/[21,22], which is a database that records the motion of macromolecules, especially proteins, from literatures of PubMed. As mentioned earlier, it was guaranteed that none of these 170 testing protein chains is homologous to the training protein chains described in the previous subsection by having a BLAST-computed sequence identity higher than 20%.

In generating the independent testing data set, we followed the procedure elaborated above for generating the training data set in order to associate each residue in the testing protein chains with a feature vector and labelled each residue as conformationally ambivalent or not based on the annotations in the MolMovDB database. In the end, the testing data set generated contains 5807 positive samples and 54823 negative samples.

### Performance metrics

In this article, the experimental results are reported with the following performance metrics, where *TP*, *TN*, *FP*, and *FN *represent the numbers of true positive, true negative, false positive, and false negative, respectively.

The *F*-score is the harmonic mean of sensitivity and precision and is a widely used metric in machine learning research for providing a balanced assessment of the performance of a predictor.

### Comparison with Boden' s predictor of protein conformational ambivalence

In this section, the performance of the hybrid predictor proposed in this article is compared with that of the predictor proposed by Boden and et. al. The approach proposed by Boden is in fact based on a predictor of protein secondary structures [23]. In Boden' s approach, if a segment of polypeptide sequence cannot be decisively classified by the predictor of protein secondary structures, then the conformation of this segment is considered to be ambivalent under different environments [24,25]. Accordingly, Boden and et. al. calculated the entropy associated with each residue in the testing chain based on the probabilities output by the predictor of protein secondary structures. If the entropy of a residue is higher than a cut-off value, then the residue is classified as being in a conformationally ambivalent region. In calculating the entropy of a residue, Boden et. al. employed two options of the predictor of protein secondary structures. With the first option, each residue in a protein chain is predicted to belong to one of the following 3 classes of protein secondary structures, alpha-helix, beta-sheet, or coil. On the other hand, with the second option, each residue is predicted to belong to one of the 8 classes of protein secondary structures defined in [19]. Table 1 summarizes the performance delivered by Boden' s 3-class predictor with the independent testing data set. In Table 1, each row corresponds to the performance delivered by the predictor under one specific cut-off value of entropy.

**Table 1:** Performance of Boden' s predictor with different cut-off values of entropy

Entropy Cuf-off	TP	FP	TN	FN	accuracy	sensitivity	specificity	precision	F-score	MCC
0.05	5803	54661	113	0	0.098	1.000	0.002	0.096	0.175	0.014
0.10	5792	54364	410	11	0.102	0.998	0.007	0.096	0.176	0.020
0.15	5703	49720	5054	100	0.178	0.983	0.092	0.103	0.186	0.079
0.20	5572	46635	8139	231	0.226	0.960	0.149	0.107	0.192	0.093
0.25	5328	43024	11750	475	0.282	0.918	0.215	0.110	0.197	0.097
0.30	4903	38576	16198	900	0.348	0.845	0.296	0.113	0.199	0.092
0.35	4468	34265	20509	1335	0.412	0.770	0.374	0.115	0.201	0.088
0.40	4009	30101	24673	1794	0.473	0.691	0.450	0.118	0.201	0.084
0.45	3584	26400	28374	2219	0.528	0.618	0.518	0.120	0.200	0.080
0.50	3142	22971	31803	2661	0.577	0.541	0.581	0.120	0.197	0.073
0.55	2702	19722	35052	3101	0.623	0.466	0.640	0.120	0.191	0.064
0.60	2254	16500	38274	3549	0.669	0.388	0.699	0.120	0.184	0.055
0.65	1730	12617	42157	4073	0.724	0.298	0.770	0.121	0.172	0.047
0.70	1100	8011	46763	4703	0.790	0.190	0.854	0.121	0.148	0.036
0.75	684	5183	49591	5119	0.830	0.118	0.905	0.117	0.117	0.023
0.80	463	3443	51331	5340	0.855	0.080	0.937	0.119	0.095	0.020
0.85	272	2238	52536	5531	0.872	0.047	0.959	0.108	0.065	0.009
0.90	142	1339	53435	5661	0.884	0.024	0.976	0.096	0.039	0.000
0.95	71	664	54110	5732	0.894	0.012	0.988	0.097	0.022	0.000

Table 2 shows the performance delivered by the proposed hybrid predictor with the independent testing data set in comparison with Boden' s predictor under the 3-class mode and the 8-class mode. As mentioned earlier, the hybrid predictor can operate in two modes, namely the *high-sensitivity *and the *high-specifinty *mode.

**Table 2:** Performance comparison between the hybrid predictor and Boden' s predictor

Predictor	TP	FP	TN	FN	accuracy	sensitivity	specificity	precision	F-score	MCC
The hybrid predictor (high-sensitivity mode)	4123	21492	33331	1684	0.618	0.710	0.608	0.161	0.262	0.189
The hybrid predictor (high-specificity mode)	2617	11682	43141	3190	0.755	0.451	0.787	0.183	0.260	0.165
Boden' s predictor (3-class mode)with entropy threshold = 0.4	4009	30101	24673	1794	0.473	0.691	0.450	0.118	0.201	0.084
Boden' s predictor (3-class mode)with entropy threshold = 0.65	1730	12617	42157	4073	0.724	0.298	0.770	0.121	0.172	0.047
Boden' s predictor (8-class mode)with entropy threshold = 0.52	2388	27895	26879	976	0.503	0.710	0.491	0.079	0.142	0.094
Boden' s predictor (8-class mode)with entropy threshold = 0.69	1198	11563	43211	2166	0.764	0.356	0.789	0.094	0.149	0.082

The numbers in Table 2 reveal that when the hybrid predictor and Boden' s predictor deliver comparable sensitivity, the hybrid predictor can deliver higher specificity and precision. Furthermore, when the hybrid predictor and Boden' s predictor deliver comparable specificity, the hybrid predictor can deliver higher sensitivity and precision.

### Comparison with Kuznetsov' s predictor of protein conformational ambivalence

In this section, the performance of the hybrid predictor proposed in this article is compared with that of the sequence-based predictor proposed by Kuznetsov [11,26]. It must be noted that Kuznetsov employed a different definition of protein conformational ambivalence. By Kuznetsov' s definition, a residue in a protein chain is said to be flexible, if its phi degree varies more than 62 or its psi degree varies more than 68 in two different conformations. In order to accommodate Kuznetsov' s definition, we labelled the residues in our collection of training protein chains based on Kuznetsov' s definition and then trained our hybrid predictor with this separately-generated training data set.

The testing data set used in this experiment was derived from Boden' s collection of testing protein chains. Again, in order to accommodate Kuznetsov' s definition of protein conformational ambivalence, we labelled the residues in the testing protein chains based on Kuznetsov' s definition. Furthermore, in order to carry out a fair comparison, we removed those testing protein chains in Boden' s collection that are homologous to one or more protein chains in Kuznetsov's training set by having a sequence identify higher than 20%. In the end, 137 out of the 170 testing protein chains in Boden' s collection were used for carrying out the benchmark reported in this section.

Table 3 reports how the hybrid predictor proposed in this article performed in comparison with Kuznetsov' s predictor [26]. The numbers in Table 3 reveal that when the parameters of Kuznetsov' s predictor and the hybrid predictor are set to deliver comparable levels of specificity, the hybrid predictor can deliver higher sensitivity and precision. It must be noted that in this experiment we carried out an additional run of cross validation to set the parameters of the hybrid predictor differently due to the fact that a different definition of conformational ambivalence is adopted. Furthermore, the results of the proposed hybrid predictor reported in Table 3 include those obtained with only the RVKDE based classifier enabled and those with both classifiers enabled. In this experiment, when both classifiers in the proposed hybrid predictor were enabled, the outputs of these two classifiers were merged based on a slightly different mechanism described in the following. In case the RVKDE based classifier predicts a residue to be conformationally ambivalent but the QUICKRBF based classifier makes an opposite prediction, then the hybrid predictor will check the predictions made by the QUICKRBF based classifier for the four adjacent residues. If any one of the four adjacent residues is marked as conformationally ambivalent, then the concerned residue will be marked as conformationally ambivalent as well. Otherwise, it will be marked as conformationally rigid. Similarly, in the case where the QUICKRBF based classifier predicts a residue to be conformationally ambivalent but the RVKDE based classifier makes an opposite prediction, then the hybrid predictor will check the predictions made by the RVKDE based classifier for the four adjacent residues. If any one of the four adjacent residues is marked as conformationally ambivalent, then the concerned residue will be marked as conformationally ambivalent as well. Otherwise, it will be marked as conformationally rigid.

**Table 3:** Performance comparison between the hybrid predictor and Kuznetsov' s predictor

Predictor	TP	FP	TN	FN	accuracy	sensitivity	specificity	precision	F-score	MCC
The hybrid predictor with only the RVKDE based classifier enabled	1236	9697	34877	1033	0.771	0.545	0.782	0.113	0.187	0.166
The hybrid predictor with both the QUICKRBF and RVKDE based classifiers enabled	813	4792	39782	1456	0.867	0.358	0.892	0.145	0.207	0.166
Kuznetsov' s predictor With false positive rate = 20	1020	9205	35369	1249	0.777	0.450	0.793	0.100	0.163	0.126
Kuznetsov' s predictor With false positive rate = 10	633	4676	39898	1636	0.865	0.279	0.895	0.119	0.167	0.118

## Discussion

**Figure 3:**
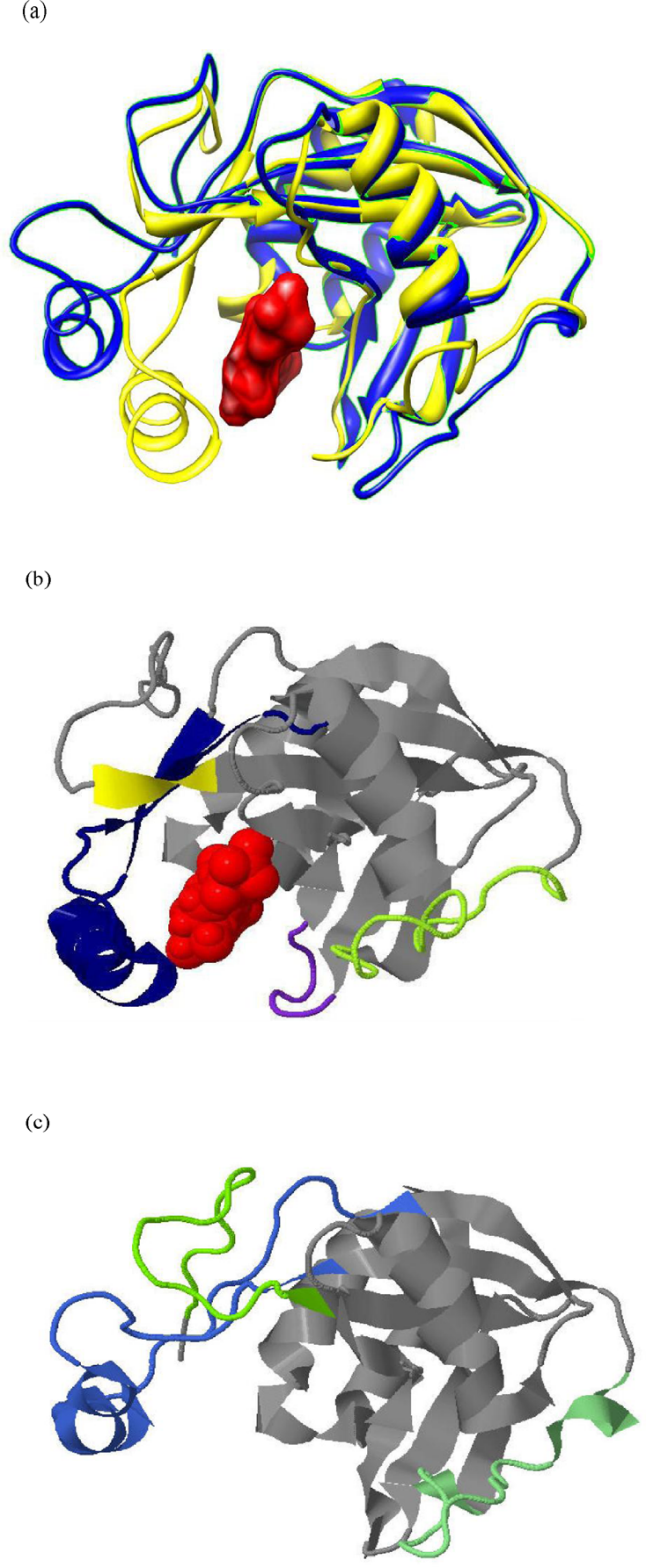
**A case study**. (a) Two conformations of protein Ap_4_A hydrolase are plotted by Chimera [36] with different colors. The one colored by yellow is with red-colored ligand ATP·MgF_x_, and the one colored by blue is without the ligand. (b) The conformationally ambivalent regions reported in [27,28] are plotted by Jmol [37] with colors yellow, blueviolet, darkblue, and greenyellow. (c) The conformationally ambivalent regions predicted by the proposed hybrid predictor are plotted by Jmol [37] with colors lawngreen, royalblue, and lightgreen.

Experimental results reported above show that the hybrid approach designed to exploit complementary prediction powers of distinctive supervised learning algorithms works more effectively than the existing predictors. In this section, we will present a real case to demonstrate the effects delivered by the proposed hybrid predictor. Fig. 3(a) depicts two conformations of protein Ap_4_A hydrolase from Lupinus angustifolius L. One conformation is with ligand ATP·MgF_x_, of which the PDB ID is 1JKN [27], and another conformation is without the ligand, of which the PDB ID is 1F3Y [28]. Fig. 3(b) and 3(c) depict the conformationally ambivalent regions identified in [27,28], and those regions predicted by the proposed hybrid predictor, respectively. It must be noted that in drawing Fig. 3(c) if a gap between two predicted conformationally ambivalent segments contains 4 or fewer residues, then the residues in the gap were also colored as if they were predicted to be in a conformationally ambivalent region. Fig. 3(a), (b), and 3(c) altogether reveal that the conformationally ambivalent regions predicted by the proposed hybrid predictor largely overlap with the structural segments that swing widely in Fig. 3(a). Furthermore, the three predicted conformationally ambivalent regions cover three out of the four conformationally ambivalent regions identified in [27,28]. Meanwhile, the only conformationally ambivalent region identified in [27,28] that does not overlap with the predicted conformationally ambivalent regions is the one with the smallest swing, which is colored by blueviolet in Fig. 3(b). As conformational transition plays a key role in carrying out several essential types of biological functions, including enzyme catalysis, macromolecule recognition, binding, and signal transduction, what the case presented in Fig. 3 demonstrates is that the hybrid predictor proposed in this article is capable of providing the biologists with valuable clues about the functional sites in a protein chain.

## Conclusion

In this article, we propose a novel approach to design a sequence-based predictor for identifying conformationally ambivalent regions in proteins. The novelty in the design stems from incorporating two classifiers based on two distinctive supervised learning algorithms that provide complementary predictive powers. Experimental results show that the overall performance delivered by the hybrid predictor proposed in this article is superior to the performance delivered by the existing predictors. Furthermore, the case study presented in this article demonstrates that the hybrid predictor proposed in this article is capable of providing the biologists with valuable clues about the functional sites in a protein chain.

Nevertheless, experimental results also show that there exists a large room for improvement with respect to the performance of the predictor. Therefore, it is of great interest to investigate how to design more advanced predictors. In this respect, it is of interest to investigate how physiochemical properties of polypeptide segments can be more effectively exploited. In this study, we have only exploited the information in the PSSM and a natural extension is to investigate the effects of incorporating the other physiochemical properties of polypeptide segments recently exploited by the related studies [29-31]. Furthermore, it is of interest to investigate the effects of incorporating the lately-developed machine learning approaches, e.g. the random forest design and the multi-stage design [32,33]. As conformational transition plays a key role in carrying out several essential types of biological functions, design of more advanced predictors deserves our continuous attention.

## Methods

### Design of the proposed hybrid predictor

**Figure 4:**
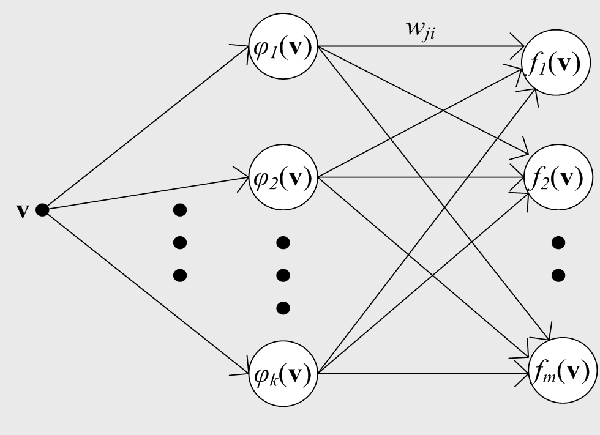
**The schematic diagram of a RBF network**.

As shown in Fig. 1, the hybrid predictor proposed in this article consists of two classifiers that are constructed with two distinctive supervised learning algorithms. The motivation to incorporate two distinctive classifiers was to exploit the complementary prediction powers of distinctive supervised learning algorithms. Fig. 4 depicts the schematic diagram of a RBF (Radial Basis Function) network for data classification applications. A RBF network consists of three layers, namely the input layer, the hidden layer, and the output layer. The input layer broadcasts the feature vector of the input query sample to each node in the hidden layer. Upon receiving an input vector, each node in the hidden layer then generates an activation based on its associated radial basis function ***ϕ***_*i*_(**v**). Finally, each node in the output layer computes a linear combination of the activations generated by the hidden nodes. The general mathematical expression of the output nodes in a RBF network with Gaussian activation functions is as follows:

where *f*_*j *_(**v**) is the function corresponding to the *j*-th output node and is a linear combination of *k *radial basis functions with center *μ*_*i *_and bandwidth *σ*_*i*_; *w*_*ji *_is the weight associated with the link between the *j*-th output node and the *i*-th hidden node. For data classification applications, the RBF network has one output node corresponding to one class of samples and a query sample is predicted to belong to the class of which the corresponding output node yields the maximum value. The tasks that the learning algorithm of a RBF network carries out include: (1) determining the centers of the activation functions associated with the hidden nodes; (2) setting the parameters associated with the activation functions; (3) optimizing the weights associated with the links between the hidden layer and the output layer.

In our implementation of the QUICKRBF package, the user can specify the number of hidden nodes to be incorporated and then the learning algorithm will place the activation functions at a set of randomly selected training samples. Our experience suggest that how a RBF network performs in terms of classification accuracy is not sensitive to how the bandwidths associated with the activation functions are set, as long as the weights in Equation (1) are optimized. Therefore, the QUICKRBF algorithm simply employs a default value and resorts to the Cholesky decomposition [34] to optimize the weights in Equation (1).

The second classifier in the proposed hybrid predictor is based on the relaxed variable kernel density estimation (RVKDE) algorithm. A kernel density estimator is in fact an approximate probability density function. Let {***s***_1_, ***s***_2_, ..., ***s***_*n*_} be a set of sampling instances randomly and independently taken from the distribution governed by probability density function *f *in the *d*-dimensional vector space. Then, with the RVKDE algorithm, the value of *f *at point **v **is estimated as follows:

where

1) ;

2) *R*(***s***_*i*_) is the maximum distance between ***s***_***i ***_and its *k *nearest training instances;

3) Γ(·) is the Gamma function [35];

4) *β *and *k *are parameters to be set either through cross validation or by the user.

For data classification applications, one kernel density estimator is created to approximate the distribution of each class of training instances. Then, a query instance located at **v **is predicted to belong to the class that gives the maximum value with the likelihood function defined as follows:

Where |*S*_*j*_| is the number of class-*j *training instances, and  (**v**) is the kernel density estimator corresponding to class-*j *training instances. In our current implementation, in order to improve the efficiency of the classifier, we include only a limited number, denoted by *k'*, of the nearest class-*j *training instances of **v **while evaluating  (**v**).

### Parameter setting

As equation (2) exhibits, there are 3 parameters, *β*, *k*, and *k' *associated with a RVKDE based classifier. Supposedly, *d *should be equal to the dimension of the feature vectors associated with the samples. However, due to the fact that there may exist correlations among features, *d *is treated as a parameter to be set during the learning process. As a result, to create a RVKDE based classifier, there are a total of 4 parameters to be set. On the other hand, to create a QUICKRBF based classifier, the user only need to determine the number of hidden nodes to be incorporated.

In order to figure out the optimal parameter settings for the proposed hybrid predictor, we have carried out the conventional 5-fold cross validation with the 3404 training protein chains described earlier. The parameters whose values were set with the 5-fold cross validation include the number of hidden nodes in QUICKRBF based classifier, parameters *β*, *k*, *k'*, and *d *associated with the RVKDE based classifier, as well as the width of the window employed to derive the feature vector of a residue from the PSSM. Table 4 and Table 5 summarize how these parameters have been set in the proposed hybrid predictor. In the experiment conducted to compare the performance of the hybrid predictor and Kuznetsov' s predictor, the parameter values shown in Table 5 were adopted because a different definition of conformational ambivalence had been employed. In this respect, the parameter values shown in Table 5 were only adopted in this particular experiment and the prediction shown in Fig. 3(c) was obtained with the parameters in the proposed hybrid predictor set in accordance with Table 4.

**Table 4:** Parameter settings of the proposed hybrid predictor for the experiment reported in Table 2

QuickRBF	RVKDE			
Number of hidden nodes	*d*	*β*	*k*	*k'*
1400	1	4	30	200

**Table 5:** Parameter settings of the proposed hybrid predictor for the experiment reported in Table 3

QuickRBF	RVKDE			
Number of hidden nodes	*d*	*β*	*k*	*k'*
1400	1	5	25	190

## Competing interests

The authors declare that they have no competing interests.

## Authors' contributions

MHY and YCL worked together to design the predictor and conducted the experiments. WLL provided many valuable suggestions for this study. CKH and YJO proposed and supervised this research. All authors have read and approved the final manuscript.

## Note

Other papers from the meeting have been published as part of *BMC Bioinformatics* Volume 10 Supplement 15, 2009: Eighth International Conference on Bioinformatics (InCoB2009): Bioinformatics, available online at http://www.biomedcentral.com/1471-2105/10?issue=S15.
